# Supplementary material for: Predicting Sensitivity to Adverse Lifestyle Risk Factors for Cardiometabolic Morbidity and Mortality
Source: Nutrients. 2022 Aug 1;14(15):3171. doi: 10.3390/nu14153171 (PMC9370461; doi:10.3390/nu14153171)
Supplement: Supplementary file 1 [file nutrients-14-03171-s001.zip › nutrients-1843386-supplementary.pdf]

# Supplementary material: Predicting sensitivity to adverse lifestyle risk factors for cardiometabolic morbidity and mortality.

## Supplementary tables

### Supplementary Table S1. VHU Criteria for exclusions on cardiometabolic traits

- Height: <130 cm or >210 cm
- Weight: <35 kg
- BMI: <15 kg/m$^2$ or >70 kg/m$^2$
- Systolic blood pressure: <20 or >300
- Diastolic blood pressure: <20 or >250
- Total cholesterol: <0.5 mmol/l or >15 mmol/l
- Triglycerides: <0.15 mmol/l or >20 mmol/l. Triglycerides values lower than 0.8 mmol/l were additionally excluded due to the sensitivity of the Reflotron benchtop analyser
- HDL-cholesterol: <0.15 mmol/l or>7 mmol/l
- LDL-cholesterol: Not defined. LDL cholesterol values lower than 0.5 mmol/l and higher than 13 mmol/l were excluded
- Fasting glucose: <1 mmol/l or >25 mmol/l. Fasting glucose values lower than 2 mmol/l were additionally excluded as they were considered biologically implausible
- *2hr glucose: <1 mmol/l or >35 mmol/l. 2hr glucose values lower than 2 mmol/l were additionally excluded as they were considered biologically implausible

* Not applied to MDCS dataset.
Note: To convert cholesterol to mg/dl multiply by 38.67, blood glucose multiply by 18.0182, triglycerides multiply by 38.67

### Supplementary Table S2. VHU Criteria for implausible values for lifestyle variables

Distance to work in kilometres (one way): All answers beyond 200 km were excluded
Grams of tobacco smoked per week: All answers equal or beyond 350 gr/week were excluded
Arachidonic acid (ARA) intake (g/day): All answers equal or beyond 0.9 gr/day were excluded
Eicosapentaenoic acid (EPA) intake (g/day): All answers equal or beyond 2 gr/day were excluded
Sodium intake (mg/day): All answers equal or beyond 10000 mg/day were excluded

### Supplementary Table S3. Variables removed during data processing.

| Trait | Variables: VHU | Variables: MDC |
|---|---|---|
| Fasting glucose | - | *gcehfn* |
| Diastolic blood pressure | *sm_duration.1; sm_duration.2; sbt.1;.2* | *systolic* |
| 2hr glucose | *ssn_time.1;sn_time.2 ;sm_status2.1 ;sm_status2.2* | |
| High-density lipoprotein cholesterol (HDL-C) | - | *kole_s* |
| Low-density lipoprotein cholesterol (LDL-C) | *FA160_sum1.1; FA160_sum1.2; MONOsum1.1; MONOsum1.2; mfetsum1.1; mfetsum1.2; FA140_sum1.1; FA140_sum1.2; fettsum1.1; fettsum1.2; FA170_sum1.1; FA170_sum1.2* | *f140_s* |
| Body mass index (BMI) | *sn_time.1;sn_time.2; sm_num_cig.1; sm_num_cig.2; FA226_sum1.1; FA226_sum1.2; karosum1.1; karosum1.2; kolhsum1.1; kolhsum1.2; protsum1_anim.1; protsum1_anim.2 ; sm_duration.1; sm_duration.2; fettsum1.1; fettsum1.2; Folasum1.1; Folasum1.2; MONOsum1.1; MONOsum1.2 ;FA160_sum1.1; FA160_sum1.2; kolesum1.1* | *gcehfn, gberrtf, rbapot, gpotdfri, rfrpotdi, rfrpoul, rbopoul, gfishm, rpifish, rcosandw,rwasandw* |
| Systolic blood pressure | *dbt.1; dbt.2* | *diastolic* |
| Total cholesterol | *FA160_sum1.1;FA160_sum1.2; FA226_sum1.1; FA226_sum1.2; karosum1.1; karosum1.2; mfetsum1.1;mfetsum1.2; POLYsum1.1; POLYsum1.2;fettsum1.1; fettsum1.2; Folasum1.1; Folasum1.2; MONOsum1.1;* | *f140_s, f204_s* |

| | | |
|---|---|---|
| | *MONOsum1.2; NATRsum1.1 ;NATRsum1.2; NIACsum1.1; NIACsum1.2; sm_duration.1; sm_duration.2* | |
| Triglycerides | *sn_time.1; sn_time.2; karosum1.1;karosum1.2; MONOsum1.1; MONOsum1.2; sm_cig_groups.1; sm_cig_groups.2; sm_duration.1;* | *gmarglf1, gmarglf2, gmarglf3* |

| Trait | Variables: VHU | Variables: MDC |
|---|---|---|
| | *sm_duration.2; FA226_sum1.1; FA226_sum1.2* | |
| (-): Not applicable | | |

**Supplementary Table S4. VHU variable meaning**

| VHU Variable | Meaning (units) |
|---|---|
| livskvalitet_d9 | Fitness status |
| sf_3f | Physical limitation to participate in moderately demanding activities: bending down or kneeling |
| sf_3d | Physical limitation to participate in moderately demanding activities: walking up several stairs |
| sf_3a | Physical limitation to participate in strenuous activities: running, lifting heavy objects, taking part in physically demanding sports |
| sf_1 | Self-rate of overall health |
| g5 | Everyday exercise satisfaction |
| sf_3g | Physical limitation to participate in moderately demanding activities: walking more than 2 km |
| sf_11d | Excellent health |
| halsoar | Overall state of health during the last year |
| beskbltr | Informed of having high blood pressure |
| g6 | Exercise during the last three months |
| livskvalitet_d12 | Energy status |
| sf_3e | Physical limitation to participate in moderately demanding activities: walking up one flight of stairs |
| halsojf | Overall state of health compared to other your age |
| sf_7 | Pain during the last four weeks |
| sf_3b | Physical limitation to participate in moderately demanding activities: moving a table, vacuuming, walking in the forest or gardening |
| sf_8 | How much has the pain during the last four weeks disturbed your normal work? |
| pa_index_miss | Cambridge physical activity index |
| g1_3 | Cycle to work vs passive travel to work |
| sf_11b | As healthy as anyone |
| sf_9a | For how much of the time during the last four weeks have you felt really alert and strong? |
| sf_3h | Physical limitation to participate in moderately demanding activities: walking more than a few hundred meters |
| sf_4b | Physical limitation that made you do less than you wanted during the last four weeks |
| kottport | Average portion size of meat/fish |
| sf_9g | For how much of the time during the last four weeks have you felt worn out? |
| sf_9e | For how much of the time during the last four weeks have you felt Full of energy? |
| protsum1_anim | Animal based protein intake (g/day) |
| potport | Average portion size of potatoes/rice/pasta |

| VHU Variable | Meaning (units) |
|---|---|
| gramnew47 | Sausage as main dish |
| sf_3j | Physical limitation to participate in moderately demanding activities: bathing or getting dressed |
| Lig_Secsum1 | Secoisolariciresinol intake (µg/day) |
| FA204_sum1 | Arachidonic acid (ARA) intake (g/day) |
| sf_4c | Physical limitation that made you not being able to perform certain work tasks or other activities during the last four weeks |
| sf_9i | For how much of the time during the last four weeks have you felt tired? |
| NIACsum1 | Vitamin B3 intake (mg/day) |
| Lig4sumsum1 | Sum of Lariciresinol, Matairesinol, Pinoresinol, Secoisolariciresinol intake (µg/day) |
| sf_11a | Get sick more often than other people |
| sf_3i | Physical limitation to participate in moderately demanding activities: walking a hundred meters |
| fibesum1 | Fibre intake (g/day) |
| sf_4d | Physical limitation that limited your ability to perform certain work tasks or other activities during the last four weeks |
| diab_foraldrar_syskon | Parents or siblings have diabetes |
| NATRsum1 | Sodium intake (mg/day) |
| g3_a | Frequency of walking during leisure time |
| g3_b | Frequency of cycling during leisure time |
| gramnew65 | Sodas, soft drinks, juice |
| gramnew43 | Minced meat dishes |
| sjukskriven | Long-term sickness |
| g4 | Changed every day exercise during the last year |
| Lig_Pinsum1 | Pinoresinol intake (µg/day) |
| Folasum1 | Folate (µg/day) |
| gramnew46 | Bacon |
| Lig_Larsum1 | Lariciresinol intake (µg/day) |
| gramnew34 | Fried potatoes, pommes frites |
| Dsum1 | Vitamin D intake (µg/day) |
| g7 | If you exercise, change in exercise habits during the last year |
| sf_3c | Physical limitation to participate in moderately demanding activities: lifting or carrying grocery bags |
| scorning | Participation in sports or physical exercise associations |

| VHU Variable | Meaning (units) |
|---|---|
| protsum1 | Total protein intake (g/day) |
| utbild | Educational level |
| sf_11c | Worsen health in the future |
| l1_4 | Porridge w/o sandwich for breakfast vs does not breakfast at all |
| MONOsum1 | Monounsaturated fat intake (g/day) |
| MOSAsum1 | Monosaccharides intake (g/day) |
| gramnew45 | Steak, chop, e.g., |
| gramnew8 | Salad dressing with oil |
| sf_6 | Extent to what your physical and emotional health disrupted your usual social life during the last four weeks |
| l1_5 | Gruel w/o sandwich for breakfast vs does not breakfast at all |
| gramnew48 | Hamburger |
| gramnew67 | Boiled coffee |
| kolesum1 | Cholesterol intake (g/day) |
| Lig_Matsum1 | Matairesinol intake (µg/day) |
| sm_status2 | Former smokers vs non-smokers |
| sf_10 | For how much of the time during the last four weeks has your physical health or your emotional problems limited your ability to interact with others? |
| gramnew44 | Meat stew |
| gramnew62 | Low fat milk (0.5%) |
| l1_3 | Sour milk, cereals, w/o sandwich for breakfast vs does not breakfast at all |
| sf_5b | Emotional problems that made you do less than you wanted during the last four weeks |
| ansttyp_a | Permanent employment |
| gramnew18 | Sausage, liver pâté on bread |
| gramnew72 | Wine |
| FULLKsum1 | Whole grain intake (g/day) |
| sf_4a | Physical limitation that reduced the normal time spent at work or in other activities during the last four weeks |
| gramnew41 | Pancake, waffle, Swedish dumpling |
| protsum1_veg | Plant based protein intake (g/day) |
| Lig_Sumsum1 | Sum of all lignans intake (µg/day) |
| kolhsum1 | Carbohydrates intake (g/day) |

| VHU Variable | Meaning (units) |
|---|---|
| Bstrsum1 | Beta-sitosterol intake (mg/day) |
| ansttyp_g | Retirement pensioner full-time |
| g3_f | Frequency of hunting or fishing during leisure time |
| B12sum1 | Vitamin B12 intake (µg/day) |
| sacksum1 | Sucrose intake (g/day) |
| gramnew31 | White cabbage, lettuce, lettuce cabbage, spinach, borecole |
| livskvalitet_d5 | Satisfaction with leisure time |
| askosum1 | Vitamin C intake (mg/day) |
| gramnew42 | Pizza |
| l5a | Eat breakfast |
| Tstrsum1 | Sum of phytosterols intake (mg/day) |
| g3_g | Frequency of picking berries or mushrooms during leisure time |
| FA160_sum1 | Palmitic acid intake (g/day) |
| selesum1 | Selenium intake (µg/day) |
| Sstrsum1 | Stigmasterol intake (mg/day) |
| socforening_e | Participation in other association |
| gramnew6 | Margarine for cooking |
| gramnew24 | Fibre cereals |
| gramnew30 | Tomato, cucumber |
| Lig_Equsum1 | Equol intake (µg/day) |
| alkosum1 | Alcohol intake (g/day) |
| gramnew3 | Low fat margarine on bread |
| karosum1 | beta-carotene intake (mg/day) |
| g3_c | Frequency of dancing during leisure time |
| sf_5c | Emotional problems that made you do be less thorough than usual in work or other activities during the last four weeks |
| fettsum1 | Total fat intake (g/day) |
| gramnew68 | Tea |
| MAGNsum1 | Magnesium intake (mg/day) |
| sf_2 | Self-rate of overall health compared to a year ago |

| VHU Variable | Meaning (units) |
|---|---|
| arbtala | Possibility to speak with colleagues during breaks |
| gramnew73 | Liquor, spirits |
| sn_quantity | Number of snuff boxes per week |
| sf_5a | Emotional problems that reduced the normal time spent at work or in other activities during the last four weeks |
| B6sum1 | Vitamin B6 intake (mg/day) |
| gramnew29 | Root vegetable tables, carrot |
| sf_9h | For how much of the time during the last four weeks have you felt happy? |
| g2_c | Light and physically active work |
| gramnew66 | Brewed (filtered) coffee |
| sf_9c | For how much of the time during the last four weeks have you felt so depressed that nothing could cheer you up? |
| livskvalitet_d10 | Appetite status |
| sm_num_cig | Number of cigarettes smoked per day |
| gramnew26 | Berries (fresh or frozen) |
| g1_2 | Walk to work vs passive travel to work |
| gramnew33 | Boiled or baked potato |
| FA205_sum1 | Eicosapentaenoic acid (EPA) intake (g/day) |
| sn_time | Years using snuff |
| sf_9f | For how much of the time during the last four weeks have you felt gloomy and sad? |
| sm_duration | Years smoking |
| g2_a | Sedentary or standing work |
| FA226_sum1 | Docosahexaenoic acid (DHA) intake (g/day) |
| l1_2 | Coffee/tea and wheat buns or rusk for breakfast vs does not breakfast at all |
| gronport | Average portion size of vegetables |
| gramnew7 | Oil for cooking |
| gramnew12 | White (soft) bread, thin crisp bread |
| civil3 | Marital status: Single vs Divorced/separated |
| Lig_Syrsum1 | Syringaresinol intake (µg/day) |
| l2 | Eat lunch |
| skiftarbete | Work shifts/weekends |

| VHU Variable | Meaning (units) |
|---|---|
| gramnew64 | Milk, sour milk (3%) |
| socforening_b | Participation in study circles |
| j10 | Suggestions that you drink less |
| ansttyp_h | Retirement pensioner part-time |
| l5b | Eat lunch |
| l1_1 | Coffee/tea and sandwich for breakfast vs does not breakfast at all |
| g9 | Amount of exercise during the last 12 months |
| gramnew69 | Light beer |
| sf_9d | For how much of the time during the last four weeks have you felt calm and serene? |
| gramnew55 | Salty fish |
| gramnew38 | Pasta |
| gramnew20 | Oatflake, whole wheat, rye or barley porridge |
| gramnew22 | Sour milk, yoghurt (3% fat) |
| g10 | Time spent in a week in moderately strenuous activities |
| sn_status2 | Former snuff users vs non-snuff users |
| FA170_sum1 | Heptadecanoic acid intake (g/day) |
| livskvalitet_d13 | Patience status |
| ansttyp_d | Unemployment |
| ZINCsum1 | Zinc intake (mg/day) |
| Cstnsum1 | Campestanol intake (mg/day) |
| j2 | Amount of alcohol drunk in a day |
| arbfort | Job demands to work very fast |
| gramnew32 | Mixed frozen vegetables |
| gramnew70 | Medium beer |
| gramnew16 | Soft cheese |
| Bstnsum1 | Beta-sitostanol intake (mg/day) |
| gramnew35 | Mashed potato |
| j3 | Frequency of drinking six or more glasses at the same occasion |
| retisum1 | Vitamin A intake (mg/day) |

| VHU Variable | Meaning (units) |
|---|---|
| antal_km | Distance to work in kilometres (one way) |
| sleep_h8a | Snore during sleep |
| j1 | Frequency of alcohol consumption |
| hjartinf_foraldrar_syskon | Parents or siblings had a cerebral haemorrhage/thrombosis or cardiac infarction before the age of 60 |
| gramnew59 | Sugar, honey, marmalade, jam |
| livskvalitet_d6 | Hearing status |
| mfetsum1 | Saturated fat intake (g/day) |
| g1_4 | Irregular travel mode to work vs passive travel to work |
| livskvalitet_d15 | Sleep status |
| sleep_h7b | Risk of sleeping while watching TV |
| gramnew23 | Sour milk, yoghurt (low fat) |
| livskvalitet_d4 | Satisfaction with economy |
| j8 | Times during last year that you drink so much that you were not able to remember what you did |
| gramnew40 | Blota (broth + bread) |
| arbkontakt | Frequent social contacts with colleagues during work |
| livskvalitet_d3 | Satisfaction with work situation |
| FA150_sum1 | Pentadecanoic acid intake (g/day) |
| gramnew25 | Corn flakes |
| DISAsum1 | Disaccharide intake (g/day) |
| soclago | Would you say that the number of people that you meet in your everyday life is enough or would you like to meet more or fewer people? |
| arbrut | Repetitive job |
| sm_gr_tobacco | Grams of tobacco smoked per week |
| soctrost | Receive hugs to comfort and support you |
| gramnew61 | Chips, popcorn, salted nuts |
| FOSFsum1 | Phosphate intake (mg/day) |
| livskvalitet_d11 | Mood status |
| gramnew27 | Apple, pear, peach, orange, mandarin and grapefruit |
| gramnew17 | Soft whey cheese |
| Lig_Endsum1 | Enterodiol intake (µg/day) |

| VHU Variable | Meaning (units) |
| --- | --- |
| gramnew9 | Cream, creme fraiche, sour cream |
| sleep_h7g | Risk of sleeping while sitting still after having lunch |
| jernsum1 | Iron intake (mg/day) |
| gramnew60 | Cookies, pastry |
| gramnew28 | Banana |
| Lig_Medsum1 | Medioresinol intake (µg/day) |
| sleep_h7f | Risk of sleeping while sitting and talking with someone |
| gramnew58 | Sweets |
| sleep_h7c | Risk of sleeping while sitting inactive in a public place |
| j6 | Times during last year that you felt you needed a drink after drinking the day before |
| gramnew19 | Meat on bread |
| gramnew2 | Butter on bread |
| sn_status1 | Snuff user vs non-snuff user |
| socsam | Number of social interactions during a normal week |
| l3 | Eat dinner |
| gramnew49 | White meat (poultry) |
| socstod | Support from others |
| B2sum1 | Vitamin B2 intake (µg/day) |
| g3_e | Frequency of gardening during leisure time |
| livskvalitet_d1 | Satisfaction with home and family situation |
| l1_0 | Only coffee/tea for breakfast vs does not breakfast at all |
| TRANSsum1 | Trans fat intake (g/day) |
| JODIsum1 | Iodine intake (µg/day) |
| Lig_Enlsum1 | Enterolactone intake (µg/day) |
| sambo2 | Cohabitation: Live alone vs Only children |
| graviditetsdiabetes | Had gestational diabetes |
| sochem | Number of friends that can come to your home at any time and feel at home |
| socdelta | Participation in associations or voluntary organizations |
| sleep_h7e | Risk of sleeping while lying down resting in the afternoon |

| VHU Variable | Meaning (units) |
|---|---|
| livskvalitet_d14 | Confidence status |
| i1 | Teetotaler |
| j4 | Times during last year that you felt inability to stop drinking |
| gramnew63 | Milk, sour milk (1,5%) |
| gramnew56 | Smoked fish/meat |
| ensum1 | Total energy intake (kcal/day) |
| POLYsum1 | Polyunsaturated fat intake (g/day) |
| sleep_h7h | Risk of sleeping in a car which has stopped for a few minutes |
| Cstrsum1 | Campesterol intake (mg/day) |
| gramnew5 | Butter for cooking |
| socupps | Appreciation of the ones at home or others |
| livskvalitet_d16 | Do you feel important and appreciated outside your home? |
| livskvalitet_d2 | Satisfaction with accommodation |
| arbski | Skill demand from job |
| gramnew14 | Cheese 28% |
| sleep_h7a | Risk of sleeping while sitting and reading |
| FA140_sum1 | Formic acid intake (g/day) |
| sleep_h7d | Risk of sleeping as a passenger in a car for one hour without break |
| ansttyp_c | Work at home |
| FA182_sum1 | Linoleic acid intake (g/day) |
| arbvad | Control over own work assignment |
| gramnew10 | Whole grain crisp bread |
| gramnew15 | Cheese 10-17% |
| g2_b | Light but partly physically active work |
| gramnew4 | Margarine on bread |
| i3 | Receive critics about your alcohol consumption |
| sambo1 | Cohabitation: Live alone vs Only one adult (spouse, partner) |
| ansttyp_f | Self-employed |
| livskvalitet_d17 | Do you feel important and appreciated in your home? |

| VHU Variable | Meaning (units) |
|---|---|
| arbhin | Enough time for job assignments |
| i2 | Feel the need to reduce alcohol consumption |
| i4 | Feel uneasy or guilty because of your way of drinking |
| socforening_d | Participation in choir |
| gramnew52 | Lean fish (e.g., perch, bass, cod) |
| gramnew13 | Coffee rolls/buns, rusk |
| gramnew1 | Bregott on bread |
| arbpsyk | High mental demand from job |
| j7 | Times during last year that you felt guilty because of your drinking |
| i5 | Drunk alcohol first thing in the morning |
| TIAMsum1 | Tiamin intake (mg/day) |
| arbhur | Control over planning and execution of the workday |
| j9 | Hurt people because of your drinking |
| arbnytt | Learn new things at job |
| gramnew37 | Rice |
| soclana | People to ask for favours from |
| soctala | Number of people with whom you can speak openly |
| sm_status5 | Former occasional smoker vs non-smoker |
| gramnew51 | Liver, kidney |
| kalcsum1 | Calcium intake (mg/day) |
| sm_status1 | Smoker vs non-smoker |
| gramnew71 | Strong beer |
| sambo4 | Cohabitation: Live alone vs Other/others |
| gramnew50 | Blood based food |
| socforening_c | Participation in theatre group |
| gramnew39 | Brown beans, pea soup |
| sockont | Number of social contacts with the same interests as you |
| sf_9b | For how much of the time during the last four weeks have you felt very nervous? |
| gramnew21 | Rosehip, sweet syrup soup |

| VHU Variable | Meaning (units) |
|---|---|
| l5c | Eat dinner |
| ansttyp_e | Student |
| gramnew57 | Ice cream |
| arbfritid | Frequency of social contacts with colleagues during leisure time |
| civil4 | Marital status: Single vs Widow/widower |
| arbfys | High physical demand from job |
| socanfo | Person to confide in |
| sambo3 | Cohabitation: Live alone vs Adult and children |
| j5 | Times during last year that something was not done due to your drinking |
| KALIsum1 | Potassium intake (mg/day) |
| socnara | Close relationship with anyone |
| socofta | Frequency of engaging in clubs, associations or study circles |
| gramnew53 | Fatty fish (e.g. herring, whitefish, salmon) |
| g2_e | Physically straining work most of the time |
| gramnew54 | Shellfish (e.g. shrimps, scallops) |
| arblamna | Possibility to leave your work for a while to speak with a colleague |
| tokosum1 | Vitamin E intake (mg/day) |
| ansttyp_b | Temporary employment |
| gramnew36 | Potato salad |
| g3_d | Frequency of shovelling snow during leisure time |
| sochelp | People to ask for help apart from the ones at home |
| sm_cig_groups | Number of cigarettes smoked per day (in groups) |
| soclyck | Special person to share feelings |
| g2_d | Sometimes physically straining work |
| arbkrav | Contradictory demands in job |
| arbbesok | Last time a colleague visited you at home |
| livskvalitet_d7 | Vision status |
| sm_num_cigar | Number of cigars smoked per day |
| gramnew11 | Whole grain soft bread |

| VHU Variable | Meaning (units) |
|---|---|
| sm_status4 | Occasional smoker vs non-smoker |
| FA183_sum1 | Linolenic acid intake (g/day) |
| arbide | Ingenuity or creativity demand from job |
| livskvalitet_d8 | Memory status |
| civil2 | Marital status: Single vs Married/partner |
| sleep_h8b | Breath-holds during sleep |
| ansttyp_i | Retirement pensioner unspecified |

**Supplementary Table S5. Rank-ordered most important variables among 9 cardiometabolic traits in VHU.**

| Variable | BMI | HDL-C | LDL-C | TC | TG | SBP | DBP | FG | 2hr G | score |
|---|---|---|---|---|---|---|---|---|---|---|
| Training or exercising during the last 3 months | 1 | 1 | 1 | 1 | 1 | 0 | 1 | 1 | 1 | 8 |
| Portion size of green vegetables | 1 | 1 | 0 | 1 | 1 | 0 | 1 | 1 | 1 | 7 |
| State of health during the last year | 1 | 1 | 0 | 0 | 1 | 1 | 1 | 1 | 1 | 7 |
| Number of cigarettes smoked per day (in groups) | 1 | 1 | 0 | 1 | 0 | 1 | 1 | 1 | 1 | 7 |
| Smokers vs non-smokers | 0 | 1 | 0 | 1 | 1 | 1 | 1 | 1 | 1 | 7 |
| Cycle to work vs passive travel to work | 1 | 1 | 0 | 1 | 1 | 1 | 0 | 0 | 1 | 6 |
| Frequency of cycling during leisure time | 1 | 1 | 0 | 1 | 1 | 0 | 0 | 0 | 1 | 5 |
| Brewed (filtered) coffee | 1 | 0 | 0 | 0 | 1 | 1 | 1 | 0 | 1 | 5 |
| Sodas, soft drinks, juice | 1 | 0 | 1 | 1 | 1 | 0 | 0 | 0 | 1 | 5 |
| Portion size of meat/fish | 1 | 0 | 1 | 1 | 0 | 1 | 0 | 0 | 1 | 5 |
| Monosaccharide intake | 1 | 0 | 0 | 1 | 1 | 0 | 0 | 1 | 1 | 5 |
| Plant protein intake | 1 | 0 | 1 | 1 | 1 | 0 | 0 | 1 | 0 | 5 |
| Former smokers vs non-smokers | 1 | 0 | 0 | 0 | 1 | 1 | 1 | 1 | 0 | 5 |
| Snuff users vs non-snuff users | 0 | 1 | 0 | 0 | 1 | 1 | 1 | 0 | 1 | 5 |
| Participation in associations, voluntary organizations | 0 | 0 | 0 | 0 | 1 | 1 | 1 | 1 | 1 | 5 |

HDL-C: High-density lipoprotein cholesterol; LDL-C: low-density lipoprotein cholesterol; HbA1c: Glycated haemoglobin; 2hr G: 2h glucose tolerance; FG: Fasting glucose; TG: Triglycerides; TC: Total cholesterol, SBP: Systolic blood pressure; DBP: Diastolic blood pressure.

**Supplementary Table S6. AUCs for each trait in VHU.**

| FRS non-laboratory-based | FRS non-laboratory-based + Sensitivity status | FRS laboratory-based risk score | FRS laboratory-based risk score + Sensitivity status | ACC/AHA risk score | ACC/AHA risk score + Sensitivity status |
|---|---|---|---|---|---|
| Total cholesterol | | | | | |
| 0.72 | 0.73 | - | - | - | - |
| SBP | | | | | |
| 0.73 | 0.74 | - | - | - | - |
| DBP | | | | | |
| 0.73 | 0.73 | - | - | - | - |
| LDL-C | | | | | |
| 0.71 | 0.76 | 0.72 | 0.75 | 0.74 | 0.76 |
| HDL-C | | | | | |
| 0.69 | 0.62 | 0.67 | 0.63 | 0.70 | 0.68 |
| BMI | | | | | |
| 0.73 | 0.74 | 0.71 | 0.73 | 0.73 | 0.74 |
| 2h glucose | | | | | |
| 0.73 | 0.74 | 0.70 | 0.71 | 0.71 | 0.71 |
| Fasting glucose | | | | | |
| 0.73 | 0.73 | 0.71 | 0.73 | 0.72 | 0.72 |
| Triglycerides | | | | | |
| 0.70 | 0.69 | - | - | - | - |

" - " it was not possible to estimate the number; HDL-C: High-density lipoprotein cholesterol; LDL-C: low-density lipoprotein cholesterol; FG: Fasting glucose; SBP: Systolic blood pressure; DBP: Diastolic blood pressure; FRS: Framingham risk score; ACC/AHA: American College of Cardiology/American Heart Association.

**Supplementary Table S7. R packages used for the analyses in the current study.**

| Step | R Package | Version |
|---|---|---|
| Variable processing | *caret* | 6.0-90 |
| Missing data imputation | *missForest* | 1.4 |
| Multicollinearity | *car* | 3.0-12 |
| Performance and model assumption | *Performance* | 0.8.0 |
| Quantile regression modelling | *quantreg* | 5.88 |
| Quantile regression forest modelling | *quantregForest ; randomForest* | 1.3-7; 4.7-1 |
| Cox modelling | *Survival* | 3.2-13 |
| Cox proportional assumption | *Rms* | 6.2-0 |
| Incidence rate ratio | *fmsb* | 0.7.2 |
| Meta-analysis | *Meta* | 5.2- |

**Supplementary Table S8. Hazard ratios and 95%CI of prediction interval categories and clinical outcomes**

| | | **CVD** | | | | | | | **T2D** | | | | | | | **CVD-mortality** | | | | | | |
|---|---|---|---|---|---|---|---|---|---|---|---|---|---|---|---|---|---|---|---|---|---|---|
| **Trait/study** | **Categories** | **n** | **Events** | ***Person-years** | **HR** | **95% (CIs)** | | ***p*** | **n** | **Events** | ***Person-years** | **HR** | **95% (CIs)** | | ***p*** | **n** | **Events** | ***Person-years** | **HR** | **95% (CIs)** | | ***p*** |
| | | | | | | | | | | ***Fasting glucose*** | | | | | | | | | | | | |
| | Ref (Neutral) | 13596 | 446 | 10854,5 | 1,00 | | | | 13566 | 186 | 10838,4 | 1,00 | | | | 13596 | 31 | 10854,5 | 1,00 | | | |
| VHU | Resilient | 261 | 11 | 193,1 | 1,21 | 0,82 | 1,80 | 0,34 | 574 | 8 | 485,1 | 0,73 | 0,18 | 2,97 | 0,67 | 261 | 0 | 193,1 | - | - | - | - |
| | Sensitive | 576 | 26 | 486,5 | 1,54 | 0,85 | 2,81 | 0,16 | 261 | 2 | 193,1 | 0,83 | 0,41 | 1,68 | 0,60 | 576 | 2 | 486,5 | 1,31 | 0,31 | 5,51 | 0,71 |
| | Ref (Neutral) | 1537 | 345 | 94,2 | 1,00 | | | | 996 | 106 | 65,3 | 1,00 | | | | 1538 | 243 | 97,5 | 1,00 | | | |
| MDC | Resilient | 70 | 9 | 4,7 | 0,48 | 0,25 | 0,94 | **3,28E-02** | 52 | 3 | 3,7 | 0,47 | 0,15 | 1,48 | 0,20 | 70 | 13 | 4,7 | 1,00 | 0,57 | 1,75 | 0,99 |

| Trait/study | Categories | CVD n | CVD Events | CVD *Person-years | CVD HR | CVD 95% (CIs) | | CVD p | T2D n | T2D Events | T2D *Person-years | T2D HR | T2D 95% (CIs) | | T2D p | CVD-mortality n | CVD-mortality Events | CVD-mortality *Person-years | CVD-mortality HR | CVD-mortality 95% (CIs) | | CVD-mortality p |
|---|---|---|---|---|---|---|---|---|---|---|---|---|---|---|---|---|---|---|---|---|---|---|
| | Sensitive | 66 | 15 | 4,1 | 1,01 | 0,60 | 1,70 | 0,96 | 32 | 6 | 1,2 | 4,81 | 2,08 | 11,12 | **2,4E-04** | 79 | 14 | 4,7 | 1,18 | 0,69 | 2,03 | 0,54 |
| ***2-h Glucose† / HbA1c*** | | | | | | | | | | | | | | | | | | | | | | |
| | Ref (Neutral) | 12233 | 392 | 10063,7 | 1,00 | | | | 12210 | 110 | 10049,4 | 1,00 | | | | 12233 | 23 | 10063,7 | 1,00 | | | |
| VHU † | Resilient | 535 | 16 | 457,9 | 0,77 | 0,47 | 1,27 | 0,31 | 535 | 8 | 457,9 | 1,42 | 0,69 | 2,91 | 0,34 | 535 | 0 | 457,9 | - | - | - | - |
| | Sensitive | 192 | 6 | 160,3 | 1,33 | 0,59 | 2,98 | 0,49 | 192 | 2 | 160,3 | 1,46 | 0,36 | 5,94 | 0,59 | 192 | 0 | 160,3 | - | - | - | - |
| | Ref (Neutral) | 1524 | 323 | 93,9 | 1,00 | | | | 1018 | 110 | 66,4 | 1,00 | | | | 1512 | 233 | 96,2 | 1,00 | | | |
| MDC | Resilient | 71 | 14 | 4,3 | 0,78 | 0,45 | 1,33 | 0,36 | 48 | 1 | 3,3 | 0,18 | 0,02 | 1,28 | 0,09 | 73 | 9 | 4,6 | 0,75 | 0,38 | 1,47 | 0,40 |
| | Sensitive | 69 | 21 | 3,9 | 1,51 | 0,96 | 2,36 | 0,07 | 30 | 2 | 1,3 | 1,03 | 0,25 | 4,20 | 0,96 | 79 | 12 | 4,6 | 1,11 | 0,62 | 2,00 | 0,72 |
| ***DBP*** | | | | | | | | | | | | | | | | | | | | | | |
| | Ref (Neutral) | 13127 | 429 | 10873,5 | 1,00 | | | | 13094 | 175 | 10851,3 | 1,00 | | | | 13127 | 33 | 10873,5 | 1,00 | | | |
| VHU | Resilient | 456 | 6 | 374,1 | 0,39 | 0,17 | 0,87 | **2,10E-02** | 456 | 4 | 374,1 | 0,94 | 0,39 | 2,28 | 0,89 | 456 | 0 | 374,1 | - | - | - | - |
| | Sensitive | 252 | 19 | 202,6 | 2,25 | 1,49 | 3,40 | **1,05E-04** | 251 | 3 | 201,7 | 0,78 | 0,25 | 2,44 | 0,67 | 252 | 1 | 202,6 | 1,36 | 0,19 | 9,99 | 0,76 |
| | Ref (Neutral) | 8337 | 1755 | 484,8 | 1,00 | | | | 1089 | 122 | 70,7 | 1,00 | | | | 8337 | 1317 | 501,1 | 1,00 | | | |
| MDC | Resilient | 339 | 64 | 19,1 | 0,92 | 0,71 | 1,18 | 0,50 | 27 | 1 | 1,8 | 0,32 | 0,04 | 2,27 | 0,25 | 339 | 59 | 19,8 | 1,05 | 0,81 | 1,37 | 0,70 |
| | Sensitive | 345 | 90 | 19,3 | 1,32 | 1,07 | 1,64 | **9,40E-03** | 45 | 3 | 2,5 | 0,83 | 0,26 | 2,62 | 0,75 | 345 | 74 | 20,1 | 1,47 | 1,16 | 1,86 | **0,001** |
| ***HDL-C*** | | | | | | | | | | | | | | | | | | | | | | |
| | Ref (Neutral) | 1855 | 90 | 1416,9 | 1,00 | | | | 1849 | 31 | 1413,3 | 1,00 | | | | 1855 | 6 | 1416,9 | 1,00 | | | |
| VHU | Resilient | 95 | 3 | 70,5 | 0,64 | 0,20 | 2,03 | 0,45 | 95 | 0 | 70,5 | - | - | - | - | 95 | 0 | 70,5 | - | - | - | - |
| | Sensitive | 91 | 5 | 66,9 | 1,22 | 0,49 | 3,01 | 0,67 | 90 | 2 | 66,1 | 1,71 | 0,40 | 7,28 | 0,47 | 91 | 1 | 66,9 | 4,68 | 0,49 | 44,92 | 0,18 |
| | Ref (Neutral) | 1515 | 329 | 94,1 | 1,00 | | | | 996 | 99 | 64,9 | 1,00 | | | | 1513 | 236 | 96,8 | 1,00 | | | |
| MDC | Resilient | 75 | 23 | 4,3 | 1,69 | 1,10 | 2,58 | **1,62E-02** | 44 | 6 | 2,3 | 2,22 | 0,96 | 5,12 | 0,06 | 76 | 13 | 4,7 | 1,39 | 0,79 | 2,44 | 0,25 |
| | Sensitive | 62 | 11 | 3,7 | 1,07 | 0,59 | 1,96 | 0,82 | 44 | 1 | 2,9 | 0,22 | 0,03 | 1,61 | 0,14 | 63 | 7 | 3,9 | 0,98 | 0,46 | 2,08 | 0,96 |
| ***BMI*** | | | | | | | | | | | | | | | | | | | | | | |
| | Ref (Neutral) | 12951 | 423 | 10640,4 | 1,00 | | | | 12921 | 161 | 10621,1 | 1,00 | | | | 13028 | 30 | 257075,9 | 1,00 | | | |
| VHU | Resilient | 636 | 19 | 557,1 | 1,18 | 0,72 | 1,94 | 0,50 | 636 | 2 | 557,1 | 0,58 | 0,14 | 2,36 | 0,45 | 638 | 0 | 13024,7 | - | - | - | - |

| | | CVD | | | | | | | T2D | | | | | | | CVD-mortality | | | | | | |
|---|---|---|---|---|---|---|---|---|---|---|---|---|---|---|---|---|---|---|---|---|---|---|
| **Trait/study** | **Categories** | **n** | **Events** | ***Person -years** | **HR** | **95% (CIs)** | | ***p*** | **n** | **Events** | ***Person -years** | **HR** | **95% (CIs)** | | ***p*** | **n** | **Events** | ***Person -years** | **HR** | **95% (CIs)** | | ***p*** |
| | Sensitive | 257 | 5 | 198,2 | 0,53 | 0,20 | 1,36 | 0,19 | 255 | 12 | 196,7 | 0,64 | 0,27 | 1,53 | 0,32 | 258 | 2 | 4940,0 | 0,93 | 0,12 | 7,01 | 0,95 |
| | Ref (Neutral) | 8217 | 1751 | 479,6 | 1,00 | | | | 1050 | 97 | 68,2 | 1,00 | | | | 8217 | 1297 | 495,3 | 1,00 | | | |
| MDC | Resilient | 399 | 61 | 23,0 | 1,04 | 0,79 | 1,37 | 0,77 | 56 | 5 | 3,8 | 2,74 | 1,00 | 7,42 | 0,05 | 399 | 68 | 23,4 | 1,57 | 1,20 | 2,06 | **0,001** |
| | Sensitive | 401 | 105 | 22,0 | 0,99 | 0,77 | 1,27 | 0,94 | 44 | 8 | 2,3 | 0,54 | 0,20 | 1,43 | 0,21 | 401 | 91 | 23,1 | 1,22 | 0,93 | 1,61 | 0,15 |
| | | | | | | | | | | | ***LDL-C*** | | | | | | | | | | | |
| | Ref (Neutral) | | 91 | 1447,2 | 1,00 | | | | 1914 | 38 | 1442,3 | 1,00 | | | | 1921 | 7 | 1447,2 | 1,00 | | | |
| VHU | Resilient | 68 | 4 | 49,8 | 1,52 | 0,56 | 4,18 | 0,41 | 67 | 1 | 49,0 | 0,82 | 0,11 | 5,99 | 0,84 | 68 | 1 | 49,8 | 2,92 | 0,33 | 25,77 | 0,34 |
| | Sensitive | 114 | 12 | 90,8 | 2,17 | 1,17 | 4,02 | **1,34E-02** | 112 | 1 | 89,9 | 0,39 | 0,05 | 2,85 | 0,35 | 114 | 2 | 90,8 | 3,99 | 0,81 | 19,70 | 0,09 |
| | Ref (Neutral) | 1443 | 299 | 89,4 | 1,00 | | | | 942 | 109 | 60,6 | 1,00 | | | | 1445 | 222 | 92,4 | 1,00 | | | |
| MDC | Resilient | 98 | 24 | 6,0 | 1,32 | 0,87 | 2,00 | 0,20 | 68 | 4 | 4,4 | 0,54 | 0,20 | 1,48 | 0,23 | 95 | 16 | 6,0 | 1,26 | 0,75 | 2,09 | 0,38 |
| | Sensitive | 91 | 25 | 5,3 | 1,58 | 1,05 | 2,39 | **2,87E-02** | 58 | 5 | 3,7 | 0,73 | 0,29 | 1,79 | 0,49 | 92 | 14 | 5,7 | 1,22 | 0,71 | 2,09 | 0,48 |
| | | | | | | | | | | | ***Total Cholesterol*** | | | | | | | | | | | |
| | Ref (Neutral) | 12637 | 403 | 10425,3 | 1,00 | | | | 12610 | 150 | 10408,0 | 1,00 | | | | 12637 | 27 | 10425,3 | 1,00 | | | |
| VHU | Resilient | 651 | 14 | 523,3 | 0,78 | 0,46 | 1,33 | 0,37 | 649 | 9 | 522,4 | 1,18 | 0,59 | 2,36 | 0,63 | 651 | 2 | 523,3 | 1,74 | 0,41 | 7,40 | 0,45 |
| | Sensitive | 466 | 39 | 405,2 | 2,23 | 1,60 | 3,10 | **2,00E-06** | 463 | 11 | 404,0 | 1,70 | 0,92 | 3,14 | 0,09 | 466 | 3 | 405,2 | 2,33 | 0,70 | 7,72 | 0,17 |
| | Ref (Neutral) | 1486 | 295 | 91,9 | 1,00 | | | | 975 | 105 | 63,2 | 1,00 | | | | 1486 | 218 | 94,4 | 1,00 | | | |
| MDC | Resilient | 92 | 26 | 5,5 | 1,70 | 1,13 | 2,54 | **1,04E-02** | 59 | 5 | 3,7 | 0,90 | 0,36 | 2,22 | 0,82 | 92 | 17 | 5,6 | 1,56 | 0,95 | 2,57 | 0,08 |
| | Sensitive | 90 | 21 | 5,3 | 1,09 | 0,69 | 1,71 | 0,72 | 53 | 5 | 3,1 | 0,84 | 0,34 | 2,09 | 0,71 | 90 | 15 | 5,5 | 0,92 | 0,54 | 1,58 | 0,77 |
| | | | | | | | | | | | ***Triglycerides*** | | | | | | | | | | | |
| | Ref (Neutral) | 10498 | 330 | 8592,6 | 1,00 | | | | 10469 | 148 | 8574,8 | 1,00 | | | | 10498 | 29 | 8592,6 | 1,00 | | | |
| VHU | Resilient | 1 | 0 | 1,1 | - | - | - | - | 1 | 0 | 1,1 | - | - | - | - | 1 | 0 | 1,1 | - | - | - | - |
| | Sensitive | 378 | 16 | 311,0 | 1,11 | 0,67 | 1,83 | 0,70 | 376 | 4 | 309,6 | 0,69 | 0,25 | 1,86 | 0,46 | 378 | 2 | 311,0 | 1,43 | 0,34 | 6,00 | 0,63 |
| | Ref (Neutral) | 1194 | 277 | 72,0 | 1,00 | | | | 767 | 80 | 48,3 | 1,00 | | | | 1194 | 202 | 74,6 | 1,00 | | | |
| MDC | Resilient | 68 | 17 | 4,2 | 1,09 | 0,66 | 1,78 | 0,75 | 41 | 0 | 2,7 | - | - | - | - | 68 | 10 | 4,3 | 0,84 | 0,44 | 1,59 | 0,58 |
| | Sensitive | 72 | 16 | 4,2 | 1,02 | 0,61 | 1,69 | 0,94 | 41 | 5 | 2,3 | 1,51 | 0,60 | 3,81 | 0,38 | 72 | 15 | 4,4 | 1,39 | 0,82 | 2,36 | 0,22 |

| | | CVD | | | | | | | T2D | | | | | | | CVD-mortality | | | | | | |
|---|---|---|---|---|---|---|---|---|---|---|---|---|---|---|---|---|---|---|---|---|---|---|
| **Trait/study** | **Categories** | **n** | **Events** | ***Person-years** | **HR** | **95% (CIs)** | | ***p*** | **n** | **Events** | ***Person-years** | **HR** | **95% (CIs)** | | ***p*** | **n** | **Events** | ***Person-years** | **HR** | **95% (CIs)** | | ***p*** |
| | | | | | | | | | | | ***SBP*** | | | | | | | | | | | |
| | Ref (Neutral) | 13021 | 408 | 10773,1 | 1,00 | | | | 12996 | 170 | 10757,8 | 1,00 | | | | 13021 | 25 | 10773,1 | 1,00 | | | |
| VHU | Resilient | 474 | 6 | 377,83 | 0,46 | 0,21 | 1,04 | 0,06 | 474,00 | 5,00 | 377,83 | 0,65 | 0,26 | 1,64 | 0,37 | 474,00 | 0,00 | 377,83 | - | - | - | - |
| | Sensitive | 201,00 | 10,00 | 162,55 | 1,63 | 0,87 | 3,05 | 0,13 | 200,00 | 5,00 | 161,41 | 1,74 | 0,71 | 4,24 | 0,22 | 201,00 | 1,00 | 162,55 | 2,48 | 0,33 | 18,46 | 0,37 |
| | Ref (Neutral) | 8236 | 1708 | 477,5 | 1,00 | | | | 1048 | 106 | 67,5 | 1,00 | | | | 8236 | 1307 | 493,1 | 1,00 | | | |
| MDC | Resilient | 357 | 61 | 21,1 | 0,87 | 0,68 | 1,13 | 0,30 | 45 | 4 | 3,0 | 0,87 | 0,32 | 2,40 | 0,79 | 357 | 56 | 21,6 | 1,01 | 0,77 | 1,32 | 0,93 |
| | Sensitive | 429 | 122 | 23,4 | 1,57 | 1,31 | 1,89 | **1,48E-06** | 51 | 9 | 3,1 | 1,59 | 0,80 | 3,17 | 0,19 | 429 | 94 | 24,8 | 1,53 | 1,24 | 1,88 | **7,87E-05** |

" - " it was not possible to estimate the number; † corresponds to VHU; *Per 100,000 person-years; SBP: systolic blood pressure; DBP: diastolic blood pressure; HDL-C: High-density lipoprotein cholesterol; LDL-C: low-density lipoprotein cholesterol; BMI: Body mass index; HbA1c: glycated haemoglobin; CVD: Cardiovascular disease. T2D: Type 2 diabetes. Adjustment included age, sex, BMI, fasting status, FFQ version, TEI, educational level and smoking status, physical activity, and alcohol intake.

## Supplementary figure

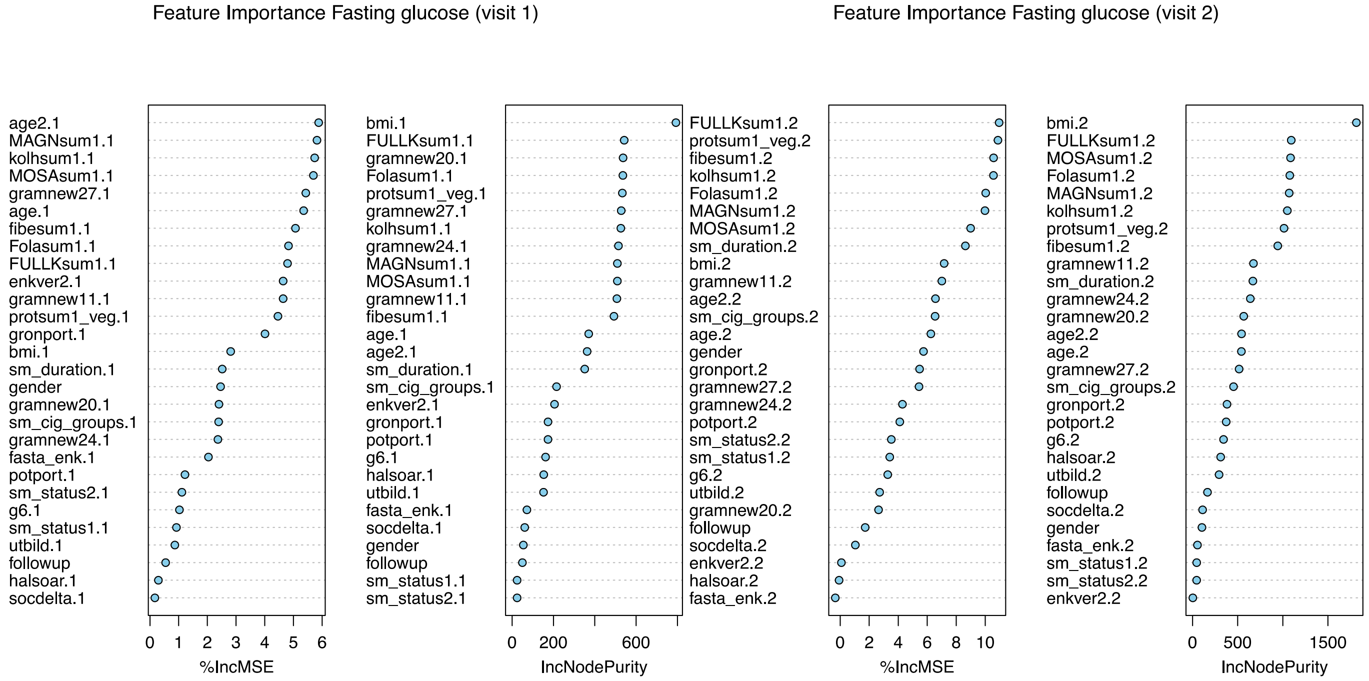


**Supplementary figure S1. Variable importance plot of fasting glucose (FG) model in VHU per visit.**
The x-axis shows each all model-variable. %IncMSE: percentage in mean square error is estimated upon mean decrease of accuracy in predictions with out-of-bag samples when each variable is excluded; IncNodePurity: increase in node purity is the total decrease of squared errors for each decision tree.

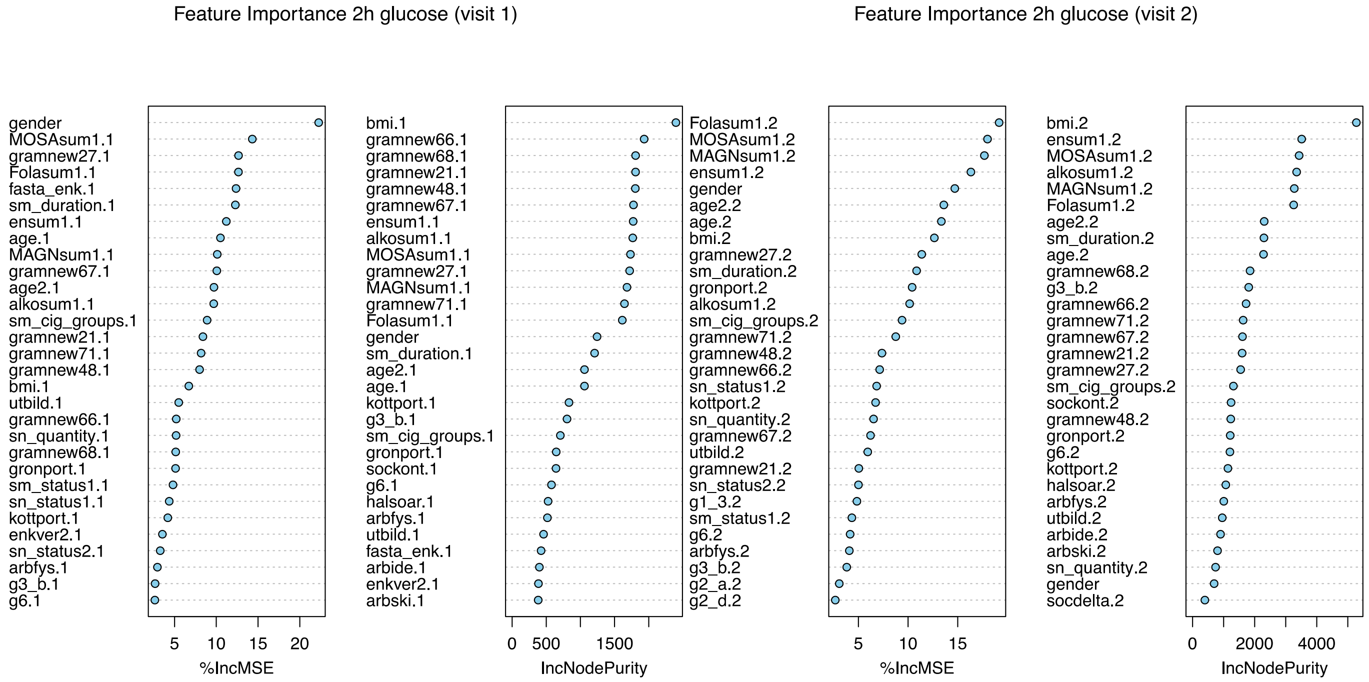


**Supplementary figure S2. Variable importance plot of 2-hour glucose (2hr G) model in VHU per visit.**
The x-axis shows each all model-variable. %IncMSE: percentage in mean square error is estimated upon mean decrease of accuracy in predictions with out-of-bag samples when each variable is excluded; IncNodePurity: increase in node purity is the total decrease of squared errors for each decision tree.

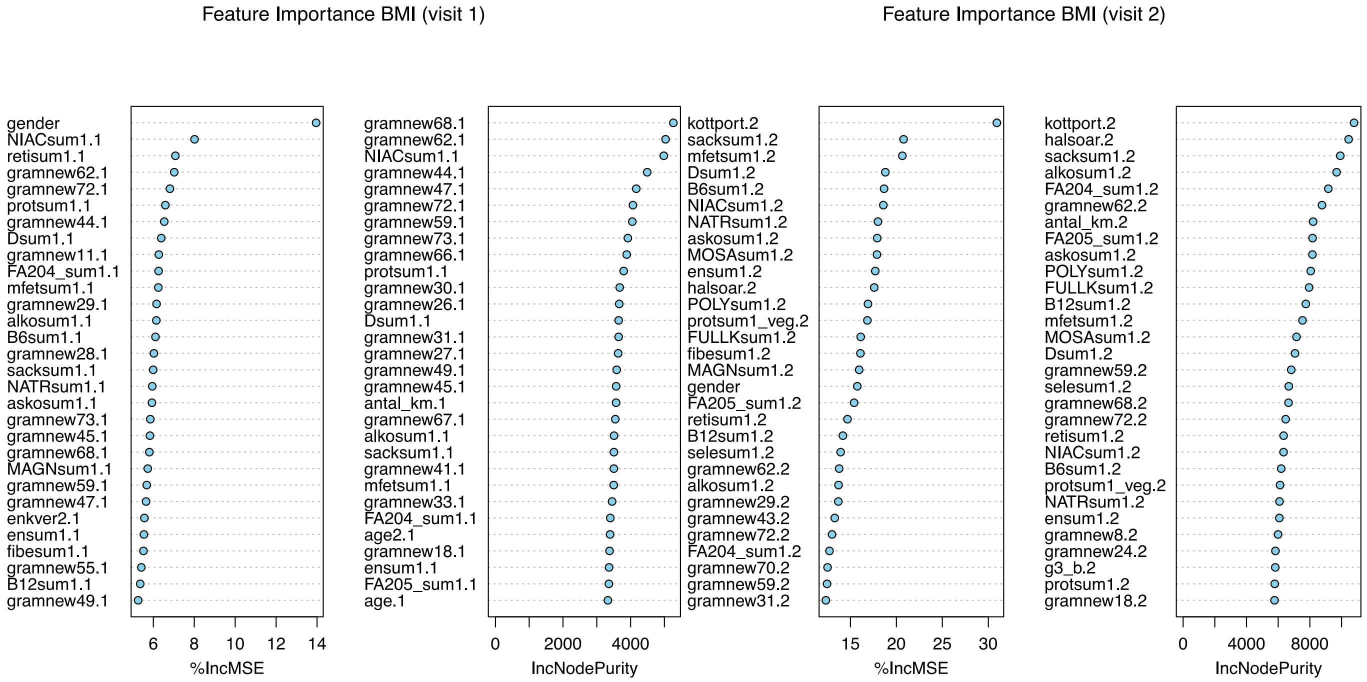


**Supplementary figure S3. Variable importance plot of body mass index (BMI) model in VHU per visit.**
The x-axis shows each all model-variable. %IncMSE: percentage in mean square error is estimated upon mean decrease of accuracy in predictions with out-of-bag samples when each variable is excluded; IncNodePurity: increase in node purity is the total decrease of squared errors for each decision tree.

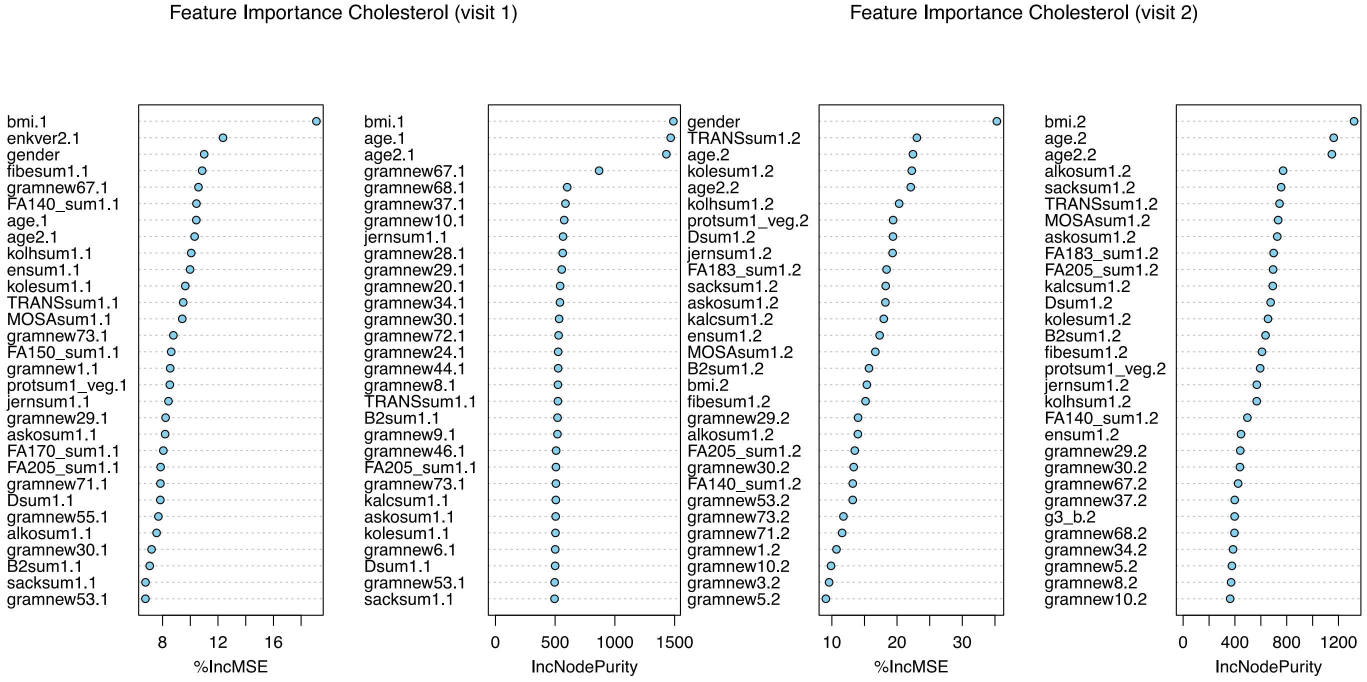


**Supplementary figure S4. Variable importance plot of Cholesterol (total cholesterol) model in VHU per visit.**
The x-axis shows each all model-variable. %IncMSE: percentage in mean square error is estimated upon mean decrease of accuracy in predictions with out-of-bag samples when each variable is excluded; IncNodePurity: increase in node purity is the total decrease of squared errors for each decision tree.

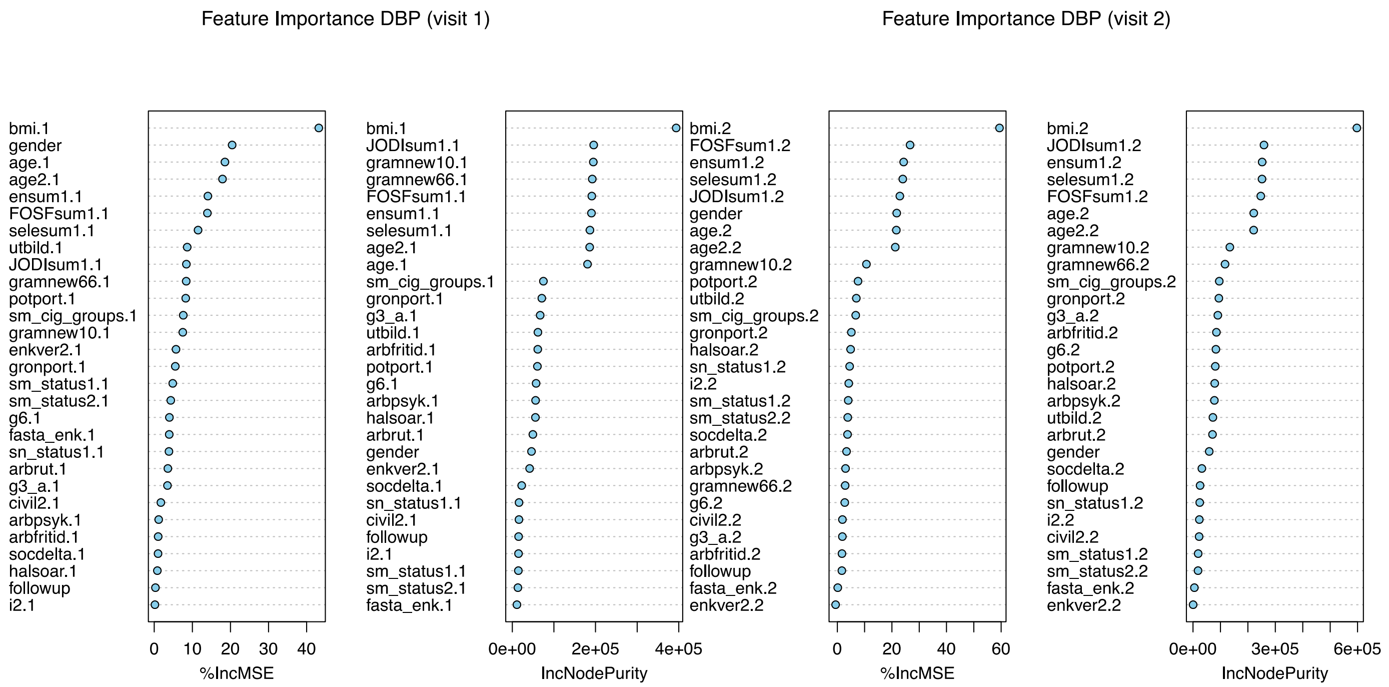

**Supplementary figure S5. Variable importance plot of diastolic blood pressure (DBP) model in VHU per visit.** The x-axis shows each all model-variable. %IncMSE: percentage in mean square error is estimated upon mean decrease of accuracy in predictions with out-of-bag samples when each variable is excluded; IncNodePurity: increase in node purity is the total decrease of squared errors for each decision tree.

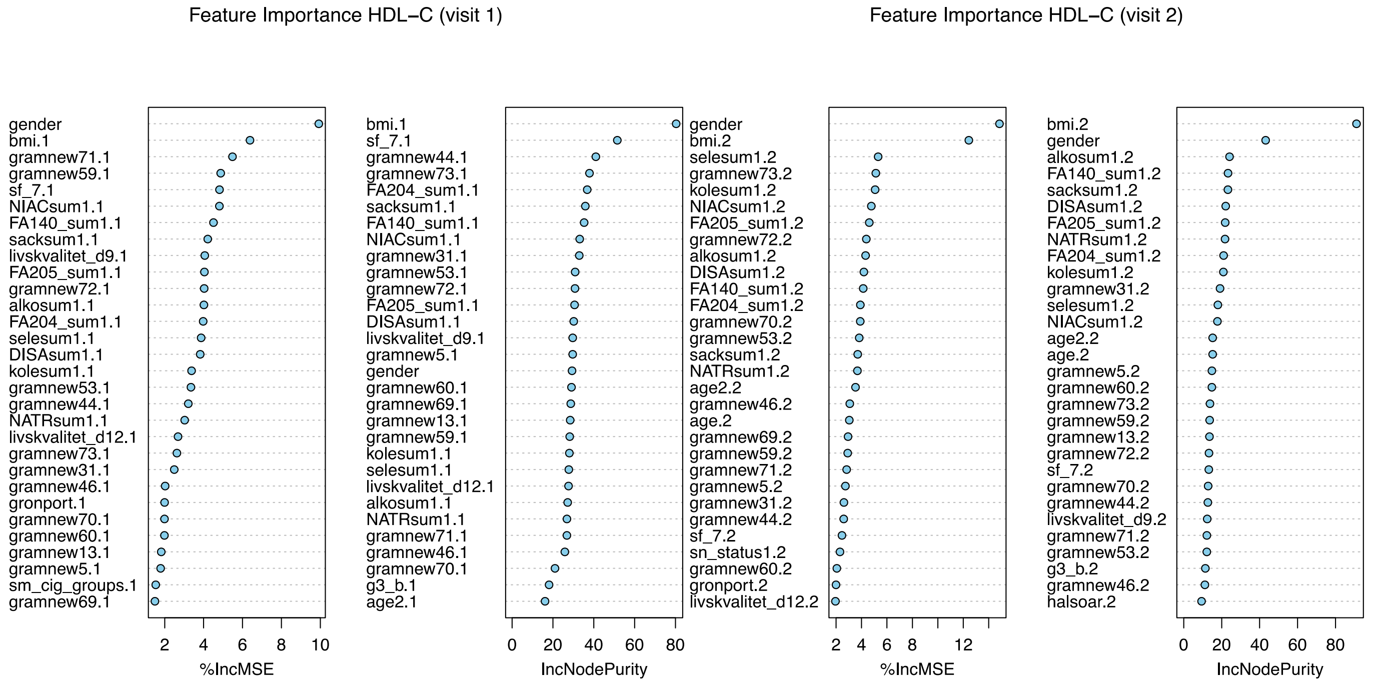

**Supplementary figure S6. Variable importance plot of high-density cholesterol (HDL-C) model in VHU per visit.** The x-axis shows each all model-variable. %IncMSE: percentage in mean square error is estimated upon mean decrease of accuracy in predictions with out-of-bag samples when each variable is excluded; IncNodePurity: increase in node purity is the total decrease of squared errors for each decision tree.

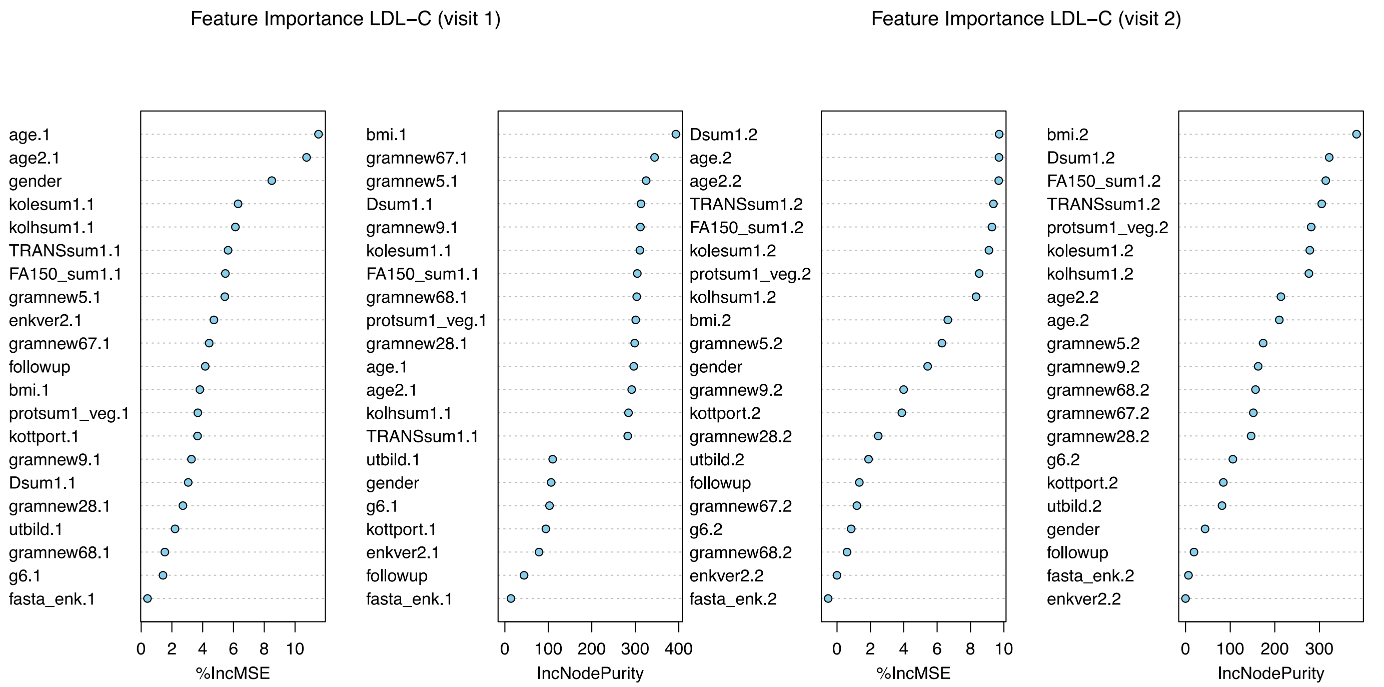


**Supplementary figure S7. Variable importance plot of low-density cholesterol (LDL-C) model in VHU per visit.**
The x-axis shows each all model-variable. %IncMSE: percentage in mean square error is estimated upon mean decrease of accuracy in predictions with out-of-bag samples when each variable is excluded; IncNodePurity: increase in node purity is the total decrease of squared errors for each decision tree.

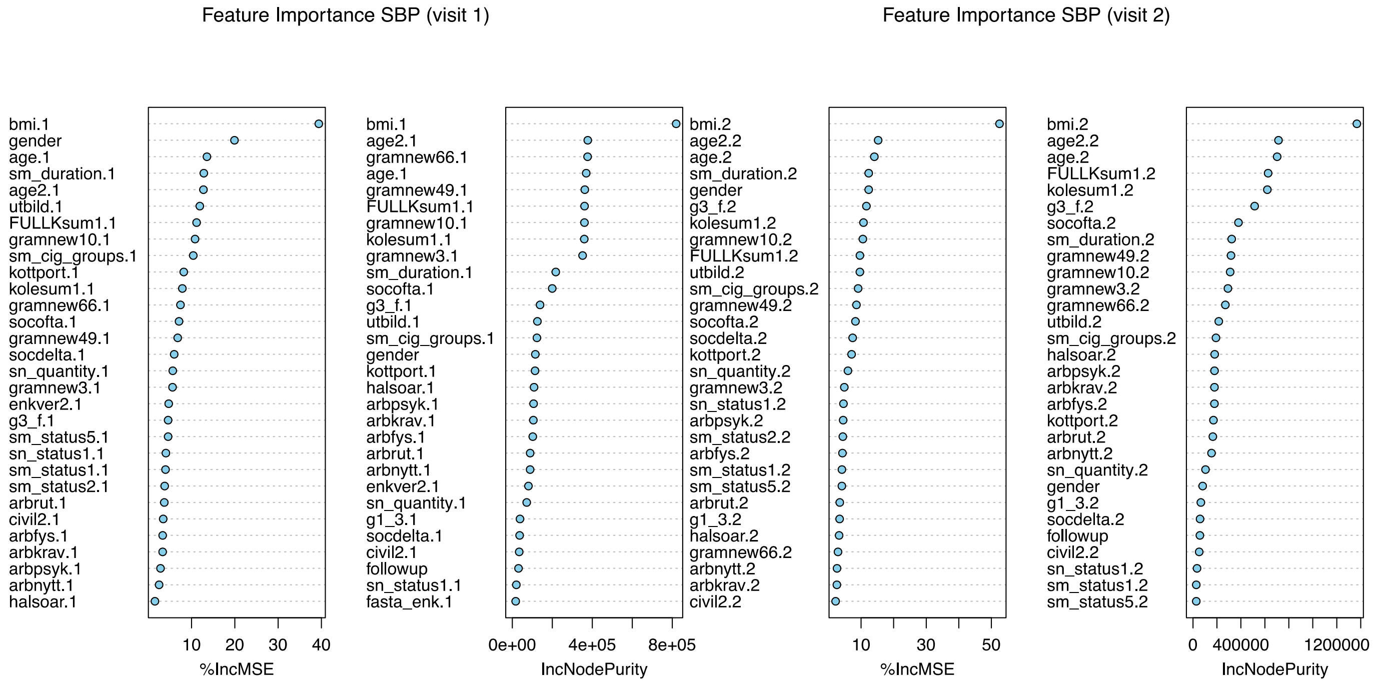


**Supplementary figure S8. Variable importance plot of systolic blood pressure (SBP) model in VHU per visit.**
The x-axis shows each all model-variable. %IncMSE: percentage in mean square error is estimated upon mean decrease of accuracy in predictions with out-of-bag samples when each variable is excluded; IncNodePurity: increase in node purity is the total decrease of squared errors for each decision tree.

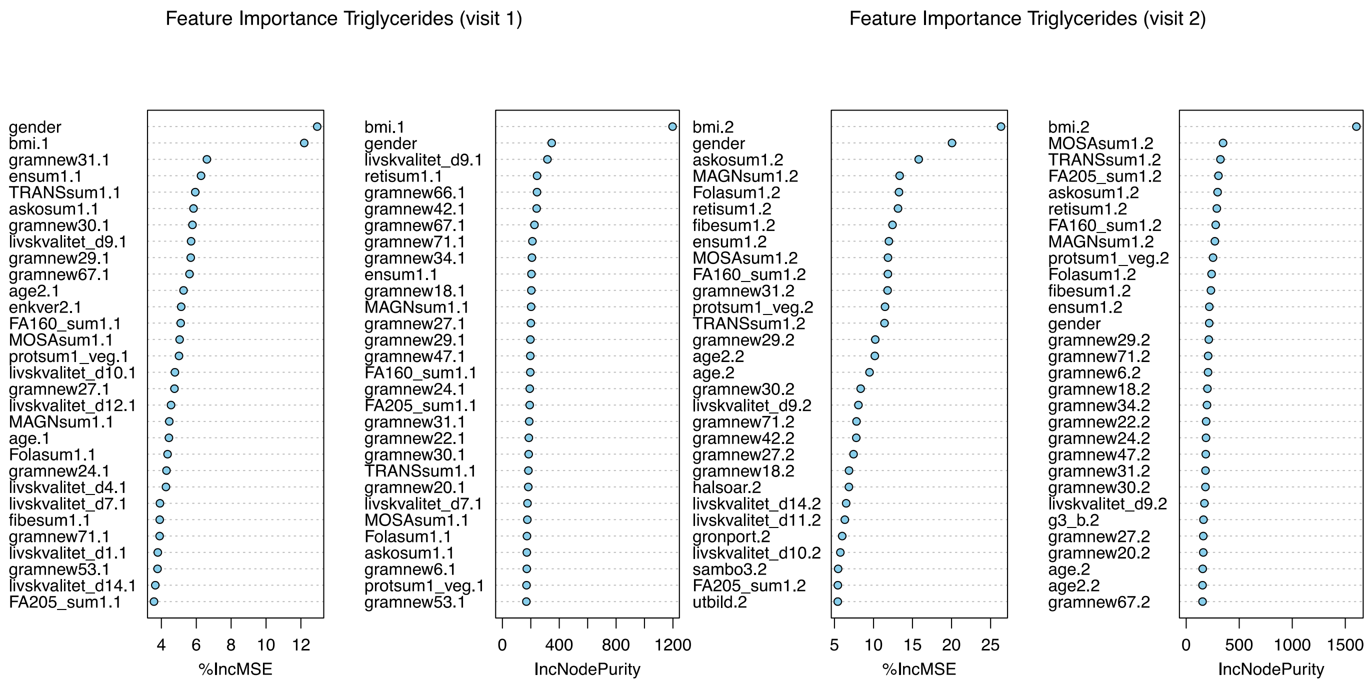


**Supplementary figure S9. Variable importance plot of triglycerides model in VHU per visit.**
The x-axis shows each all model-variable. %IncMSE: percentage in mean square error is estimated upon mean decrease of accuracy in predictions with out-of-bag samples when each variable is excluded; IncNodePurity: increase in node purity is the total decrease of squared errors for each decision tree.
